# Evodiamine Inhibits Colorectal Cancer by Downregulating ASS1 via Wnt/β-Catenin/c-MYC Pathway to Block Arginine Synthesis

**DOI:** 10.3390/ph18111736

**Published:** 2025-11-14

**Authors:** Huimin Wang, Hao Deng, Jiaming He, Jing Ma, Yunying Li, Haoyue Lv, Jianhua Ran, Dilong Chen, Jing Li

**Affiliations:** 1 Laboratory of Stem Cells and Tissue Engineering, Department of Histology and Embryology, College of Basic Medicine, Chongqing Medical University, Chongqing 400016, China; 18706941701@163.com (H.W.);; 2 The Fifth Clinical College, Chongqing Medical University, Chongqing 400016, China; 3 Laboratory of Neuroscience and Tissue Engineering, Department of Anatomy, College of Basic Medicine, Chongqing Medical University, Chongqing 400016, China; 4 Chongqing Key Laboratory of Development and Utilization of Genuine Medicinal Materials in Three Gorges Reservoir Area, Chongqing Three Gorges Medical College, Chongqing 404120, China; 5 NMPA Key Laboratory for Quality Monitoring of Narcotic Drugs and Psychotropic Substances, Chongqing Institute for Food and Drug Control, Chongqing 401120, China

**Keywords:** ASS1, evodiamine, colorectal cancer, arginine

## Abstract

**Background**: Argininosuccinate synthase 1 (ASS1), a key enzyme in arginine biosynthesis, is highly expressed in colorectal cancer (CRC) and promotes cancer progression, making it a potential therapeutic target. Evodiamine (EVO), a natural alkaloid from *Evodia rutaecarpa* acts as a novel Wnt signaling pathway inhibitor with strong anticancer activity against various cancers. However, its exact therapeutic mechanism in CRC remains unclear. **Methods**: To address this gap, experiments included enzyme-linked immunosorbent assay (ELISA) to test EVO’s effect on CRC arginine production; CCK-8, EdU, colony formation, and wound-healing assays to assess CRC cell proliferation and migration; RT-qPCR, Western blot, immunofluorescence (IF), and ShASS1 for mechanism exploration and target validation; and a syngeneic tumor allograft model to study EVO’s metabolic regulation and anticancer efficacy in CRC. **Results**: In vitro, EVO significantly inhibited arginine synthesis metabolism and reduced CRC cell proliferation/migration. In vivo, it suppressed tumor tissue arginine metabolism, slowed allograft tumor growth, and decreased ASS1 expression. Mechanistically, EVO concentration-dependently reduced ASS1 via the Wnt/β-catenin/c-MYC pathway; ShASS1 replicated EVO’s anticancer effects, confirming ASS1’s mediating role. **Conclusions**: EVO downregulates ASS1 via the Wnt/β-catenin/c-MYC pathway disrupts CRC arginine synthesis metabolism and inhibits CRC cell proliferation/migration. These results support the interaction between metabolic regulation and signaling pathways, highlighting EVO as a promising CRC therapeutic candidate.

## 1. Introduction

Colorectal cancer (CRC) is a grave form of cancer that significantly endangers human health, ranking as the third most frequently diagnosed cancer and the second most prevalent cause of cancer-related mortality [1]. Currently, chemotherapy serves as a fundamental aspect of CRC treatment. Traditional chemotherapeutic drugs, including 5-fluorouracil, irinotecan, and oxaliplatin, may hinder tumor advancement; however, their practical application is often hampered by the emergence of systemic drug resistance and toxicity in patients [2,3]. It is essential to create novel therapeutic agents that successfully integrate robust anti-tumor properties while minimizing adverse effects in current research efforts.

In recent years, bioactive compounds from traditional Chinese medicine have gained attention within oncology research for their unique benefits: they improve therapeutic effectiveness while minimizing toxicity, target various pathways, and influence metabolic processes [4,5]. One such compound is evodiamine (EVO), an indoloquinoline alkaloid obtained from the fruits of the medicinal plant *Evodia rutaecarpa*. Numerous studies have confirmed its diverse biological activities and anti-cancer properties [6]. Nevertheless, the exact mechanisms of EVO’s effects on CRC remain ambiguous, highlighting the need for further detailed investigation. A key feature of malignant tumor cells is metabolic reprogramming.

To cope with low oxygen levels and limited nutrients, tumor cells adjust their metabolic pathways by regulating cytokines and signaling cascades in the tumor microenvironment. This adaptation enables them to satisfy the increased energy and biosynthetic requirements associated with rapid cell division [7,8]. Among these metabolic adjustments, the reconfiguration of amino acid metabolism is particularly significant in tumor initiation and development [9]. Cancer cells often increase the expression of amino acid transporters or trigger synthetic pathways to obtain the biosynthetic precursors necessary for unregulated proliferation. In CRC, there is a notable rise in the endogenous production of arginine—a key component for protein synthesis and polyamine synthesis—marking a significant aspect of metabolic reprogramming. This mechanism is largely controlled by the enzyme argininosuccinate synthetase 1 (ASS1). Serving as the key enzyme that regulates the initial synthesis of arginine, ASS1 aids in the conversion of citrulline and aspartate to argininosuccinate, which subsequently transforms into arginine [10]. Research indicates that the majority of cancer types, such as melanoma, bladder cancer, myxofibrosarcoma, lymphoma, and others, demonstrate arginine auxotrophy attributed to the decreased expression of ASS1 [11,12,13,14,15,16,17,18,19]. In these cases, employing an “arginine depletion strategy” has shown efficacy in hindering tumor advancement [20]. Conversely, ASS1 is significantly overexpressed in various cancers, including CRC, where it functions as an oncogenic factor promoting both cancer cell proliferation and metastasis [21]. This implies that inhibiting ASS1 to reduce endogenous arginine production may offer a viable therapeutic approach for CRC.

Subsequent investigations have demonstrated that ASS1 expression is regulated at the transcriptional level by various signaling pathways. Notably, the Wnt/β-catenin pathway, which is commonly hyperactivated in CRC, includes c-MYC as a crucial effector molecule. c-MYC binds to the promoter region of ASS1, enhancing its transcription by modulating chromatin structure [22,23]. Based on this understanding and considering prior findings on EVO’s capacity to inhibit the Wnt pathway [24,25], we put forth the following primary hypothesis: EVO has the potential to downregulate activation of the Wnt/β-catenin/c-MYC signaling cascade, consequently leading to decreased expression of ASS1 protein. This effect would ultimately lower the synthesis of endogenous arginine in CRC cells, thereby inhibiting their proliferation and migration.

This study aims to clarify the mechanisms through which EVO influences CRC. The specific objectives comprise: (1) evaluating the effects of EVO on the synthesis and metabolism of arginine in CRC cells, as well as its role in inhibiting cell proliferation and migration; (2) investigating the modulation of ASS1 expression by EVO via the Wnt/β-catenin/c-MYC signaling pathway; and (3) confirming EVO’s regulatory effects on the growth of CRC allografts and arginine metabolism by utilizing animal models, thereby offering experimental backing for potential clinical applications.

## 2. Results

### 2.1. EVO Inhibits Arginine Synthesis Metabolism in CRC Cells

**Figure 1:**
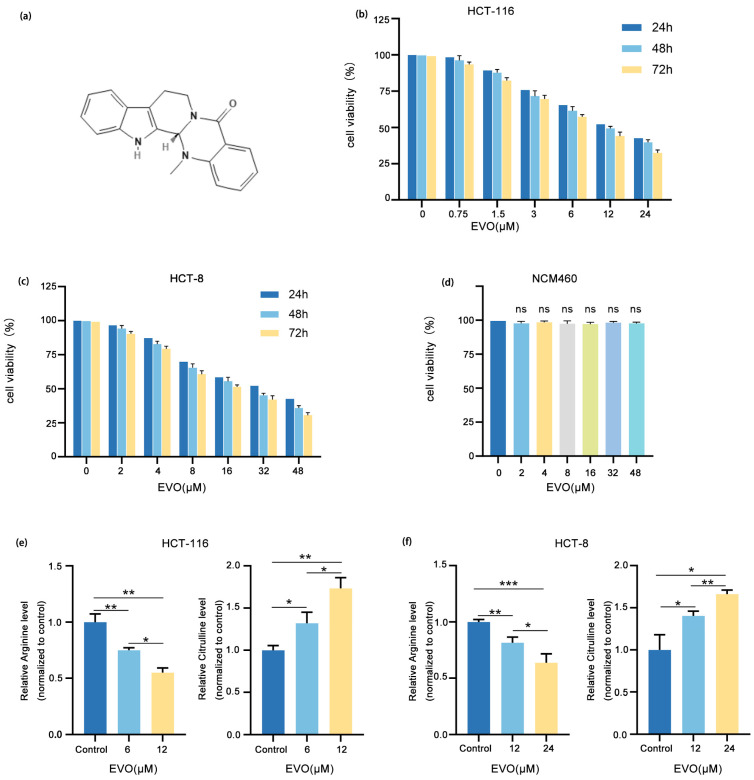
EVO Inhibits Arginine Synthesis Metabolism in CRC Cells. (**a**) Chemical structure of EVO. (**b**,**c**) The effect of EVO on the viability of HCT-116 and HCT-8 cells was evaluated by the CCK-8 assay. (**d**) The effect of EVO on the viability of NCM460 cells was evaluated by the CCK-8 assay. (**e**,**f**) The effect of EVO on their arginine synthesis metabolism of HCT-116 and HCT-8 cells were assessed by an ELISA kit. Statistical analyses used one-way ANOVA with Dunnett’s post hoc test; Data are presented as the mean ± SD, *n* = 3, ns: not statistically significant, * *p* < 0.05, ** *p* < 0.01, *** *p* < 0.001.

Figure 1a illustrates the chemical structure of EVO. Initially, HCT-116 and HCT-8 cell lines were exposed to various concentrations of EVO over periods of 24, 48, and 72 h. The CCK-8 assay was utilized to evaluate cell viability. These results demonstrated that EVO exerted a significant concentration- and time-dependent inhibitory effect on the viability of CRC cells. After 48 h of EVO treatment, the half-maximal inhibitory concentration (IC_50_) values for HCT-116 and HCT-8 cells were 11.87 μM and 23.25 μM (Figure 1b,c), respectively. Therefore, 12 μM and 24 μM were chosen as the maximum EVO concentrations for HCT-116 and HCT-8 cells in subsequent experiments, respectively. Following that, NCM460 cells underwent treatment with identical concentrations of EVO for a duration of 48 h, and CCK-8 assays indicated that EVO exerted no toxic effects on the NCM460 cells (Figure 1d). Subsequently, an ELISA was then employed to evaluate the effect of EVO on the metabolism of arginine synthesis in CRC cells (Figure 1e,f). The results showed that following 48 h of EVO treatment, arginine levels in both HCT-116 and HCT-8 cells were significantly reduced, whereas the level of citrulline (a precursor for arginine biosynthesis) was markedly increased. Collectively, these findings suggest that EVO inhibits arginine synthesis metabolism in CRC cells.

### 2.2. EVO Inhibits Proliferation and Migration in CRC Cells

**Figure 2:**
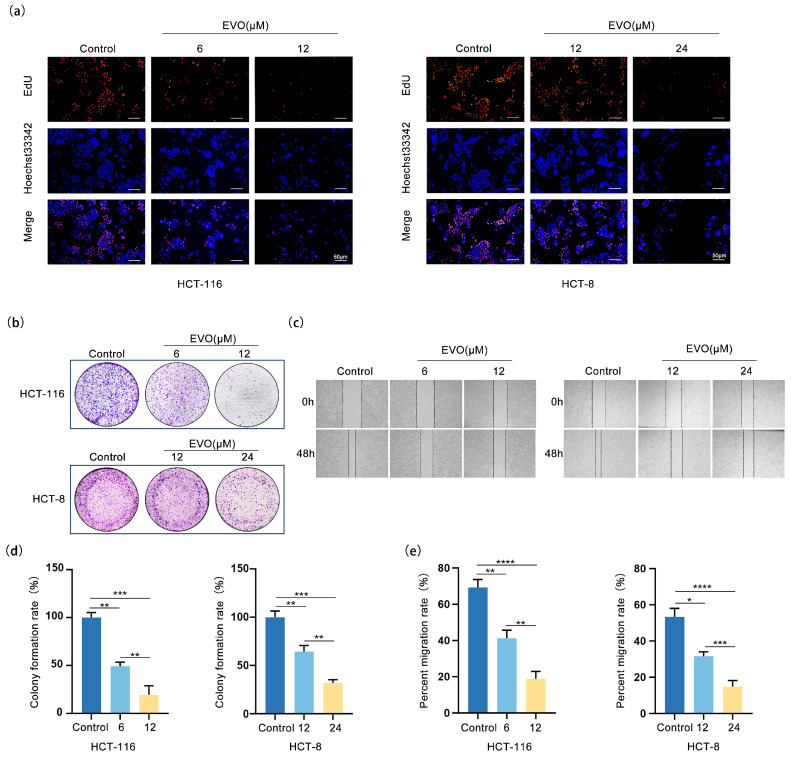
EVO Inhibits Proliferation and Migration in CRC Cells. (**a**) The effect of EVO on the proliferative capacity of HCT-116 and HCT-8 cells was evaluated by the EdU assay (Scale bar: 50 μm). (**b**,**d**) The effect of EVO on the colony formation ability of HCT-116 and HCT-8 cells was evaluated by the colony formation assay. (**c**,**e**) The effect of EVO on the migration ability of HCT-116 and HCT-8 cells was evaluated by the wound-healing assay. Statistical analyses were performed using one-way analysis of variance (ANOVA) followed by Dunnett’s post hoc test; Data are presented as the mean ± SD, *n* = 3, * *p* < 0.05, ** *p* < 0.01, *** *p* < 0.001, **** *p* < 0.0001.

To assess how EVO influences the growth and movement of the CRC cell lines HCT-116 and HCT-8, we performed EdU assay, colony formation assays, and wound-healing assays. In the EdU assay (Figure 2a), the proportion of EdU-positive (proliferating) cells in both HCT-116 and HCT-8 cultures decreased significantly with increasing concentrations of EVO in a concentration-dependent manner, confirming that EVO inhibits CRC cell proliferation. In alignment with this, assays for colony formation (Figure 2b,d) demonstrated that treatment with EVO led to a marked reduction in both the quantity and rate of colony formation in the two cell lines, suggesting a reduction in long-term proliferative capacity. Furthermore, wound-healing assays (Figure 2c,e) demonstrated that the rate of scratch closure in groups treated with EVO was notably reduced in comparison to the control group, with the inhibitory effect becoming more pronounced as EVO concentration increased, thus confirming that EVO suppresses CRC cell migration. These findings collectively indicate that EVO restricts the growth and movement of CRC cells, specifically HCT-116 and HCT-8, providing experimental evidence for further investigation into its anti-CRC mechanism of action.

### 2.3. EVO Downregulates ASS1 Upregulated in CRC Cells

**Figure 3:**
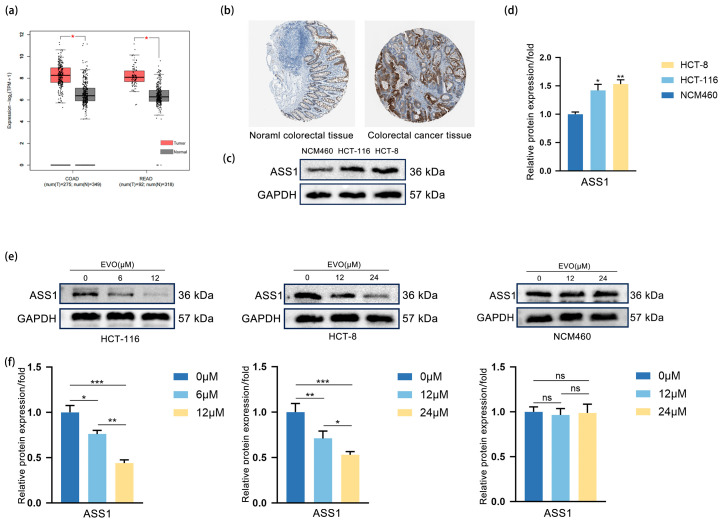
EVO Downregulates ASS1 Upregulated in CRC cells. (**a**) GEPIA analyzed ASS1 mRNA levels in CRC (COAD, READ) and normal tissues from TCGA database; (**b**) IHC (HPA database) detected ASS1 protein in normal colorectal and CRC tissues; (**c**,**d**) Western blot measured ASS1 protein in normal colon cell NCM460 and CRC cells HCT-116, HCT-8; (**e**,**f**) Western blot assessed ASS1 protein in HCT-116, HCT-8, and NCM460 cells treated with different EVO concentrations. Data are presented as the mean ± SD, *n* = 3. Statistical analyses were performed using two-tailed Student’s *t*-test (two groups) or one-way ANOVA with post hoc tests (multiple groups); ns: not statistically significant, * *p* < 0.05, ** *p* < 0.01, *** *p* < 0.001.

In order to examine the expression level of ASS1 in CRC and EVO’s regulatory effect on it, we conducted the following experiments. Analysis of the TCGA database using GEPIA (Figure 3a) revealed that the levels of ASS1 mRNA were considerably elevated in CRC tissues, including colon adenocarcinoma (COAD) and rectum adenocarcinoma (READ), compared to the nearby normal tissues. Immunohistochemical analysis (IHC) sourced from HPA database (Figure 3b) demonstrated enhanced staining of the ASS1 protein in CRC tissues relative to normal colorectal tissues. Western blotting (Figure 3c,d) demonstrated that ASS1 protein was highly expressed in CRC cell lines HCT-116 and HCT-8, but low in normal colon cell line NCM460. Upon treatment of HCT-116, HCT-8, and NCM460 with escalating levels of EVO, Western blot analysis (Figure 3e,f) demonstrated a concentration-dependent reduction in ASS1 protein levels in both HCT-116 and HCT-8, whereas NCM460 exhibited no notable change. These results suggest that EVO downregulates ASS1 expression specifically in CRC cells. Collectively, ASS1 is highly expressed in HCT-116 and HCT-8, and EVO can regulate its expression

### 2.4. EVO Regulates ASS1 via the Wnt/β-Catenin/c-MYC Pathway in CRC Cells

**Figure 4:**
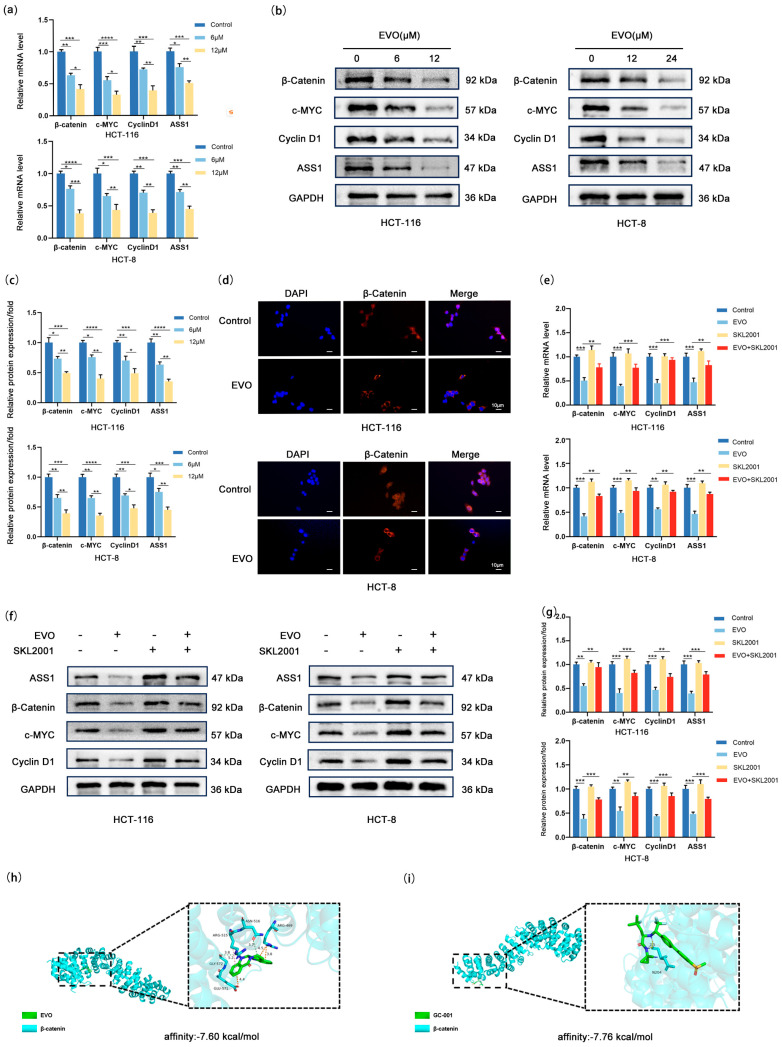
EVO Regulates ASS1 via the Wnt/β-Catenin/c-MYC Pathway in CRC Cells. (**a**) RT-qPCR detected mRNA levels of β-catenin, c-MYC, Cyclin D1, and ASS1 in HCT-116 and HCT-8 cells treated with EVO; (**b**,**c**) Western blot measured protein levels of β-catenin, c-MYC, Cyclin D1, and ASS1 in EVO-treated HCT-116 and HCT-8 cells; (**d**) Immunofluorescence staining observed subcellular localization of β-catenin in EVO-treated HCT-116 and HCT-8 cells (Scale bar: 10 μm); (**e**–**g**) RT-qPCR and Western blot analyzed mRNA and protein levels of β-catenin, c-MYC, Cyclin D1, and ASS1 in cells co-treated with EVO and SKL2001; (**h**) Three-dimensional visualization of EVO docking with β-catenin; (**i**) Three-dimensional visualization of β-catenin docking with its positive inhibitor GC-001; Data are mean ± SD, *n* = 3. Statistical analyses used two-tailed Student’s *t*-test or one-way/two-way ANOVA with post hoc tests; * *p* < 0.05, ** *p* < 0.01, *** *p* < 0.001, **** *p* < 0.0001.

In order to investigate whether the molecular mechanism by which EVO downregulates ASS1 is linked to the Wnt/β-catenin/c-MYC pathway, firstly, we detected the expression changes of key molecules in this pathway as well as ASS1. RT-qPCR (Figure 4a) showed that with increasing EVO concentrations, the mRNA levels of β-catenin, c-MYC, Cyclin D1, and ASS1 demonstrated a decrease in a concentration-dependent manner in both HCT-116 and HCT-8 cells, suggesting that EVO may regulate the activity of this pathway and ASS1 expression at the transcriptional level. Western blotting (Figure 4b,c) further verified this trend: after EVO treatment, the protein levels of β-catenin, c-MYC, Cyclin D1, and ASS1 in the two cell lines were reduced in a concentration-dependent manner. This initially suggested that EVO may downregulate the expression of downstream molecules and ASS1 by inhibiting Wnt/β-catenin/c-MYC pathway activity. To clarify the binding affinity between EVO and β-catenin, a core molecule of the Wnt/β-catenin pathway, we performed molecular docking analysis. The results showed that the binding energy of EVO to β-catenin was −7.60 kcal/mol (Figure 4h), while that of GC-001, a positive inhibitor of β-catenin, to β-catenin was −7.76 kcal/mol (Figure 4i). The similar binding energies suggest that EVO has favorable spontaneous binding affinity with β-catenin, providing molecular-level support for EVO to directly act on β-catenin and regulate the Wnt pathway. The movement of β-catenin into the nucleus is a crucial phase in the activation of the Wnt signaling pathway. Next, we observed its subcellular localization using immunofluorescence staining (Figure 4d). β-catenin accumulated significantly in the nucleus of control group cells; however, after EVO treatment, the nuclear fluorescence intensity of β-catenin was significantly reduced, with more β-catenin distributed in the cytoplasm. This confirmed that EVO can inhibit β-catenin nuclear translocation. To confirm the important function of the Wnt/β-catenin/c-MYC pathway in EVO-mediated regulation of ASS1, we performed a rescue experiment using SKL2001—a specific agonist of this pathway. Consistent findings from RT-qPCR (Figure 4e) and Western blotting analyses (Figure 4f,g) clearly indicated that treatment with SKL2001 alone notably increased the mRNA and protein levels of β-catenin, c-MYC, Cyclin D1, and ASS1. In contrast, when cells were co-treated with SKL2001 and EVO, SKL2001 effectively reversed EVO’s inhibitory effect on aforementioned molecules, restoring their expression levels to near-normal. In summary, EVO inhibits Wnt/β-catenin pathway activation (by reducing β-catenin nuclear translocation), downregulates c-MYC transcription and expression, weakens the binding and activating effect of c-MYC on the ASS1 promoter, and ultimately reduces ASS1 expression.

### 2.5. ASS1 Knockdown Synergizes with EVO to Inhibit Arginine Synthesis and Malignant Phenotypes in CRC Cells

**Figure 5:**
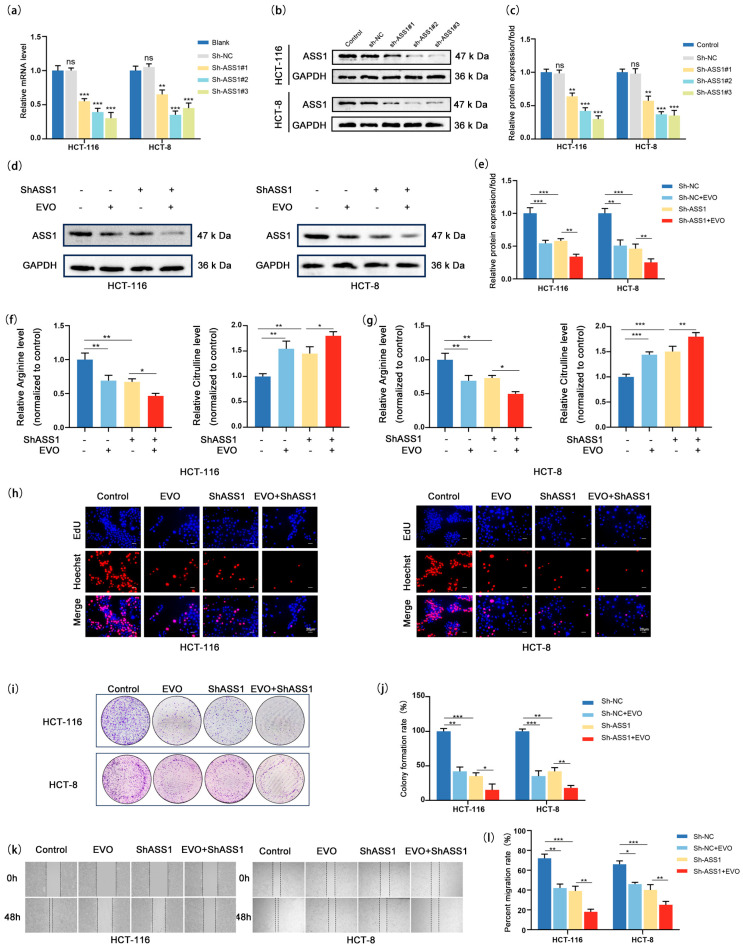
ASS1 Knockdown Synergizes with EVO to Inhibit Arginine Synthesis and Malignant Phenotypes in CRC Cells. (**a**) RT-qPCR detected ASS1 mRNA in HCT116/HCT-8 cells transduced with ShNC/ShASS1#1-3; (**b**,**c**) Western blot and quantification assessed ASS1 protein after knockdown; (**d**,**e**) Western blot and quantification measured ASS1 protein in four groups; (**f**,**g**) Intracellular arginine and citrulline levels in four groups were detected to evaluate arginine synthesis; (**h**) EdU assay showed cell proliferation (Scale bar: 20 μm); (**i**,**j**) Colony formation assay and quantification analyzed clonogenicity; (**k**,**l**) Wound healing assay and quantification assessed migratory ability. Data are mean ± SD, *n* = 3. Statistical analyses used two-tailed Student’s *t*-test or one-way/two-way ANOVA with post hoc tests; ns: not statistically significant, * *p* < 0.05, ** *p* < 0.01, *** *p* < 0.001.

Given that EVO downregulates ASS1 expression via the Wnt/β-catenin/c-MYC pathway, to further clarify the critical role of ASS1 in EVO-mediated anti-CRC effects, we first established an ASS1 knockdown model in CRC cell lines, validated the efficiency of gene silencing at both mRNA and protein levels (Figure 5a–c), and selected ShASS1#1 for subsequent experiments. Next, we set up four different groups: the control group (ShNC), the EVO-treated group, the ASS1 knockdown group, and the combined ASS1 knockdown plus EVO treatment group (EVO + ShASS1) to investigate the impact of combined intervention on ASS1 expression, arginine synthesis, proliferation, and migration. Western blotting results (Figure 5d,e) showed that when EVO was combined with ShASS1, the mRNA and protein levels of ASS1 were even lower compared to the EVO-only treatment group, indicating that superimposing ASS1 knockdown on EVO treatment could more significantly inhibit ASS1 expression. Since ASS1 is a key enzyme in arginine synthesis, we detected intracellular levels of arginine as well as citrulline, a precursor in arginine biosynthesis (Figure 5f,g): either EVO treatment alone or ASS1 knockdown alone significantly decreased arginine synthesis; moreover, the arginine level in the combined treatment group was further reduced compared to the EVO-only group, suggesting that ASS1 knockdown could synergistically enhance EVO-mediated inhibition of arginine synthesis. In cell proliferation assays, EdU assay (Figure 5h) showed that the percentage of EdU-positive cells in EVO-only treatment group was markedly reduced when compared to the control group; the ASS1 knockdown group also exhibited significantly reduced proliferative capacity; and in the group receiving combined treatment, the rate of proliferation was additionally reduced in comparison to the group treated exclusively with EVO. Consistent results were obtained from colony formation assays (Figure 5i,j). either EVO or ASS1 knockdown alone reduced the number and area of colonies formed, with the inhibitory effect being more pronounced in the combined treatment group, confirming that ASS1 knockdown could synergistically enhance EVO-mediated inhibition of cell proliferation. Wound healing assays (Figure 5k,l) showed that both EVO-only treatment and ASS1 knockdown alone significantly inhibited migration ability; the healing rate observed in the combined treatment group was even lower compared to that of the EVO-only group, indicating a synergistic effect in inhibiting cell migration. Collectively, these findings demonstrate that EVO inhibits CRC cell functions by downregulating ASS1 expression, and further knocking down ASS1 on this basis can more significantly reduce arginine synthesis and enhance the inhibition of cell proliferation and migration by exerting an additive effect with EVO-mediated suppression of the Wnt/β-catenin/c-MYC pathway along with a greater reduction in ASS1 levels. This result not only validates that ASS1 is a key downstream target for EVO’s anti-CRC effects but also provides experimental evidence for improving therapeutic efficacy through the combined targeting of ASS1 and EVO.

### 2.6. EVO Inhibits Arginine Synthesis in CRC in Vivo

**Figure 6:**
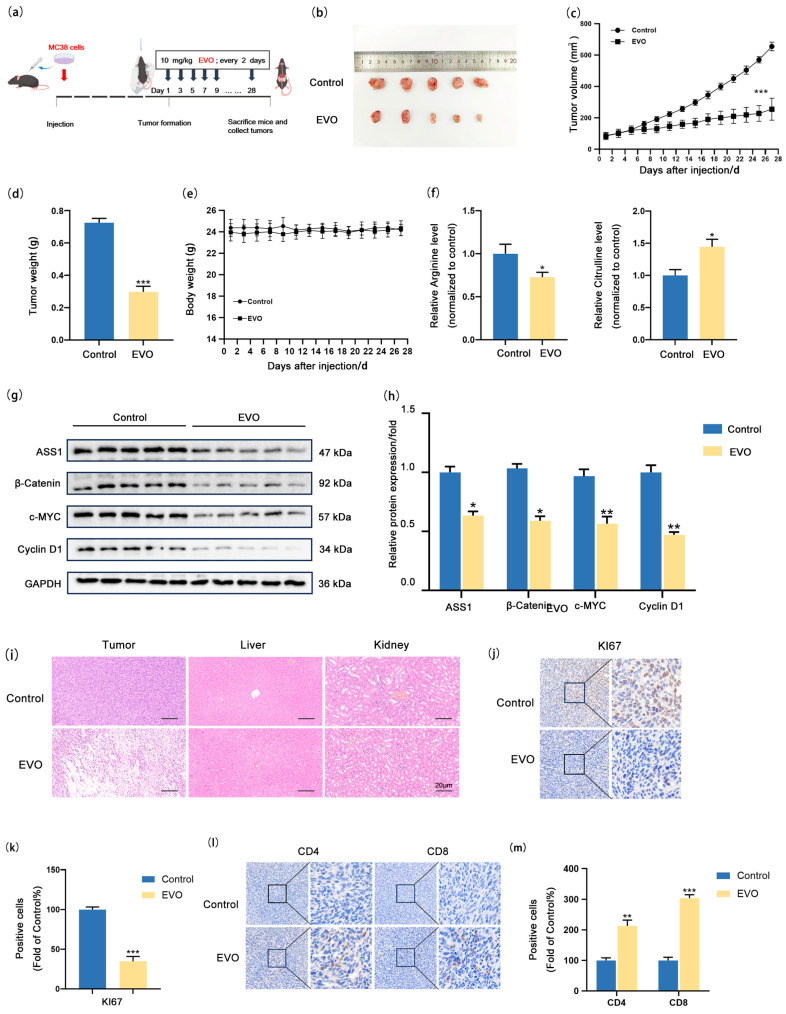
EVO Inhibits Arginine Synthesis in CRC in vivo. (**a**) Syngeneic tumor allografts were established by subcutaneously implanting MC38 cells into C57BL/6J mice; (**b**) Images of tumor allografts; (**c**) Tumor volume changes, (**d**) tumor weight, (**e**) body weight; (**f**) ELISA detected arginine and citrulline levels in tumor tissues; (**g**,**h**) Western blot measured ASS1, β-catenin, c-MYC, and Cyclin D1 protein in tumor tissues; (**i**) H&E staining of tumor, liver, and kidney tissues (Scale bar: 20 μm); (**j**,**l**) IHC detected Ki67 expression and CD4^+^/CD8^+^ T cell infiltration in tumor tissues; (**k**,**m**) Quantification of Ki67 and T cell infiltration. Data are mean ± SD, *n* = 5. Statistical analyses used two-tailed Student’s *t*-test or one-way/two-way ANOVA with post hoc tests; * *p* < 0.05, ** *p* < 0.01, *** p < 0.001.

In order to examine the in vivo effect of EVO on CRC, we established a syngeneic tumor allograft model using MC38 cells (Figure 6a) Schematic of establishing syngeneic tumor allograft model. (Figure 6b–d). Analysis of mouse body weight statistics (Figure 6e) revealed that both groups displayed a steady weight gain trend over time with no significant differences, indicating that EVO had no notable impact on the overall growth status of the mice. The EVO group demonstrated a notable reduction in the expression levels of Ki67 within the tumor tissues. (Figure 6j,k), demonstrating that EVO exerts a marked inhibitory effect on CRC syngeneic tumor allografts. ELISA results of tumor tissues (Figure 6f) indicated that EVO also significantly inhibits arginine synthesis metabolism. Western blotting analysis of tumor tissues (Figure 6g,h) found that EVO remarkably reduced the expression levels of proteins β-catenin, c-MYC, Cyclin D1, and ASS1 in subcutaneous CRC syngeneic tumor allografts. Additionally, Hematoxylin–eosin staining (HE staining) of paraffin sections from the liver and kidney of mice in both the control and EVO groups (Figure 6i) showed no obvious morphological differences under the microscope, suggesting that EVO does not exhibit notable toxic side effects in vivo. Finally, immunohistochemical staining (Figure 6l,m) was employed to assess the ratio of immune cells infiltrating the tumor in syngeneic tumor allografts, revealing a notable rise in the counts of CD4^+^ and CD8^+^ T cells in EVO-treated mice, which indicates that EVO enhances T cell infiltration in tumors. In summary, these results demonstrate that EVO is capable of suppressing the expression of the ASS1 protein via the Wnt/β-catenin/c-MYC signaling pathway, thereby suppressing arginine synthesis metabolism and tumor growth, while also activating the immune microenvironment of tumor cells.

## 3. Discussion

CRC, recognized as one of the most common types of malignant tumors globally, is distinguished by its hidden onset, significant invasiveness, and unfavorable prognosis [26]. Clinical data indicate that a majority of patients receive their diagnosis at a later stage, thereby losing the optimal opportunity for surgical treatment [27]. Additionally, existing chemotherapeutic agents are often associated with severe toxic side effects and drug resistance. Therefore, identifying novel natural-derived anti-tumor agents with high efficacy and low toxicity, alongside elucidating their mechanisms of action holds significant clinical value for improving the current status of CRC treatment.

Accumulating studies have explored the anti-tumor mechanisms of EVO in CRC, with most focusing on its regulation of signaling pathways to induce tumor cell apoptosis and inhibit growth. For instance, it has been reported that EVO enhances chemosensitivity in CRC by targeting RRM2 [28] and inhibits CRC growth through RTKs-mediated PI3K/AKT/p53 signaling pathway [29]. However, the regulatory role of EVO in tumor metabolic reprogramming, especially its crosstalk with signaling pathways, remains insufficiently investigated. Distinct from previous studies, the present work is the first to elucidate that EVO exerts anti-CRC effects through a novel mode combining signaling pathway regulation and arginine metabolism modulation.

The use of Chinese herbal monomers to interfere with metabolic reprogramming for anti-tumor purposes is a current research focus [30]. Metabolic reprogramming itself has been widely recognized as a novel target for tumor cell metabolism. It is not a fixed pattern but exhibits dynamic changes depending on tumor type and microenvironment, involving multiple metabolic pathways [31]. This phenomenon is universally observed in the initiation and progression nearly all tumors, making amino acid metabolic reprogramming a key area of investigation in cancer therapy research. Literature reports have demonstrated the following: Curcumin can downregulate the expression and activity of hexokinase II (HKII) through the phosphorylation of serine/threonine kinase (AKT), inhibit glycolysis, and induce the dissociation of HKII from mitochondria to further promote cell apoptosis [32]. Oridonin can reduce the expression levels of SREBP1 and Fas, inhibit cellular fatty acid (FA) synthesis, and induce apoptosis in SW480 and SW620 cells [33]. Physapubescin I, isolated from *Physalis pubescens* L., can target kidney-type glutaminase (KGA) to disrupt glutamine metabolism, and thereby exert an anti-tumor effect [34]. In prostate cancer cells, the paclitaxel derivative docetaxel exerts a synergistic effect with polyethylene glycol arginine deiminase (ADI-PEG20), leading to arginine depletion and subsequent tumor suppression [35]. Notably, these studies do not merely explore individual metabolic pathways in isolation but gradually reveal the multi-dimensional regulatory effects of natural products on tumor metabolic reprogramming. Whether it is blocking key steps in glycolysis, interfering with the activity of key enzymes in lipid synthesis, or inhibiting glutamine catabolism, the underlying mechanism essentially involves disrupting the “metabolic addiction” of tumor cells to curb their malignant progression. These studies further suggest that amino acid metabolism—particularly arginine metabolism—as a critical branch of tumor metabolic reprogramming has become an important direction in investigating the anti-tumor mechanisms of natural products. This direction holds special significance in CRC: Arginine serves as a foundational compound for producing several bioactive substances, such as nitric oxide (NO), ornithine, and polyamines, which include putrescine, spermidine, and spermine [36]. Abnormally enhanced endogenous arginine synthesis is a typical feature of metabolic reprogramming. Given that ASS1 functions as the rate-limiting enzyme in the synthesis of arginine de novo, it is not only highly expressed in CRC but also directly associated with the proliferative activity and invasive capacity of tumor cells [21], providing a key node for precise intervention targeting CRC metabolism.

In this study, we found that EVO exerts its inhibitory effect on CRC not by relying on traditional pro-apoptotic or anti-inflammatory pathways but by specifically targeting the arginine metabolic pathway. Experiments demonstrated that EVO significantly inhibits the growth and movement of CRC both In vitro and In vivo, consistent with conclusions from other researchers that “EVO has anti-CRC potential” [37,38,39]. Furthermore, In vitro experiments confirmed that EVO significantly reduces intracellular arginine levels in CRC cells, and this effect is highly synchronized with the downregulation of ASS1 mRNA and protein expression. The findings imply that the suppression of the malignant characteristics of CRC cells by EVO may be closely related to the regulation of ASS1-mediated arginine synthesis, pointing to a core direction for subsequent molecular mechanism research.

In-depth mechanism analysis and molecular target validation revealed that the Wnt/β-catenin/c-MYC signaling pathway is the key upstream pathway through which EVO downregulates ASS1. It is well established that aberrant activation of this pathway is a core driver of CRC development, resulting in the movement of β-catenin into the nucleus and the subsequent activation of target genes including c-MYC [40,41,42], and this activation promotes tumor proliferation and metabolic reprogramming. In contrast, EVO can downregulate c-MYC expression by inhibiting the movement of β-catenin into the nucleus, thereby reducing the transcriptional (mRNA) and protein levels of ASS1. Previous studies also confirmed via the JASPER database that the potential transcription factor c-Myc can localize to the promoter region of ASS1 and directly promote the transcriptional activation of ASS1; in contrast, when c-MYC is depleted, the upregulation of ASS1 expression is significantly suppressed [23]. This fully aligns with our observation that the expression of ASS1 shows a simultaneous decrease following EVO-mediated downregulation of c-MYC, further confirming that EVO inhibits the transcriptional activation and protein expression of ASS1 in CRC by suppressing the Wnt/β-catenin/c-MYC axis. Since ASS1 is the rate-limiting enzyme in arginine synthesis, its reduction impairs arginine synthesis in CRC cells, ultimately inhibiting tumor proliferation and migration. Further experiments combining ASS1 knockdown (ShASS1) with EVO treatment confirmed that in contrast to the EVO-only cohort, the group receiving combined treatment showed a marked reduction in arginine levels, a lower rate of EdU positivity, and diminished colony formation in CRC cells. This not only identifies ASS1 as a core target of EVO in regulating arginine metabolism but also demonstrates that the combination of EVO and targeted ASS1 inhibition can enhance anti-tumor effects by synergistically reducing arginine synthesis. Meanwhile, this provides a new perspective for understanding CRC metabolic reprogramming and experimental evidence for overcoming the limitations of single-drug therapy.

In vivo experiments further verified the clinical translational potential of the aforementioned mechanism. This study utilized a syngeneic MC38 CRC allograft model established in immunocompetent C57BL/6 mice. This model retained intact innate and adaptive immune responses, more accurately reflecting the tumor microenvironment characteristics of clinical CRC patients and systematically validated the core value and unique advantages of EVO in anti-CRC therapy. On one hand, EVO significantly inhibited allograft growth with a concurrent marked reduction in intratumoral arginine levels. Additionally, the “Wnt/β-catenin/c-MYC-ASS1” regulatory axis observed In vitro was recapitulated in the In vivo microenvironment with normal immune function, indicating that this mechanism can function stably in physiological settings with intact immunity. On the other hand, EVO exhibited favorable biosafety as mice showed steady weight gain and no morphological abnormalities in the liver or kidney and no abnormalities in liver or kidney function, overcoming the limitation of “high efficacy accompanied by high toxicity” associated with traditional chemotherapy. A notable finding was that EVO simultaneously promoted the infiltration of intratumoral CD4^+^ and CD8^+^ T cells, inducing a synergistic immune activation effect. The regulatory mechanism of the arginine-spermidine metabolic axis provides a plausible explanation for this phenomenon. It is known that arginine is a critical precursor for spermidine synthesis in tumor cells. Spermidine produced by tumor cells can inhibit the clustering of T cell receptors (TCRs) on the membranes of CD8^+^ T cells through the regulation of cholesterol metabolism, thereby impairing the anti-tumor capacity of CD8^+^ T cells [43]. Additionally, studies have indicated that excessively high arginine concentrations themselves can exert adverse effects on T cell function. For example, they can disrupt the cytoskeletal structure of CD4^+^ T cells through myosin regulation, ultimately suppressing the proliferation and migration of CD4^+^ T cells [44]. This implies that in the tumor microenvironment, not only does spermidine interfere with immune cell function but also abnormally elevated arginine itself acts as a barrier to CD4^+^ T cell infiltration. When EVO reduces arginine synthesis by inhibiting ASS1, it not only indirectly decreases intratumoral spermidine production to relieve the inhibition of CD8^+^ T cells but also alleviates the adverse effects of high arginine levels on the cytoskeletal structure and migratory capacity of CD4^+^ T cells. Simultaneously, it reduces the microenvironmental stress caused by arginine metabolic disorders, creating more favorable conditions for the infiltration and survival of CD4^+^ T cells. The discovery of this immune regulatory phenomenon not only provides direct In vivo experimental evidence for the dual anti-tumor effects of EVO (metabolic intervention and immune activation) but also offers new experimental clues for understanding how arginine metabolism regulation simultaneously affects the function of different T cell subsets and reshapes the tumor immune microenvironment.

This study confirms that EVO inhibits the Wnt/β-catenin/c-MYC signaling pathway, which results in a decrease in ASS1 expression (the enzyme that limits the rate of arginine synthesis), leading to a reduced availability of intracellular arginine and ultimately hindering the proliferation and movement of CRC cells. This finding not only reveals a novel mechanism of action for EVO but also provides experimental evidence for “signal-metabolism” combined targeted therapy for CRC.

This study also has the following limitations. First, due to difficulties in obtaining tissue samples, clinical CRC samples were lacking. Second, In vitro experiments were conducted in an ideal nutrient environment, which did not fully simulate the competitive relationship between arginine and other amino acids In vivo tumor microenvironment, potentially underestimating the actual effect of EVO. Furthermore, this study has not clearly verified whether the regulatory effect of EVO on arginine metabolism is independent of non-specific cytotoxicity, that is, it has not ruled out the possibility that this metabolic regulatory effect is secondary to decreased cell viability. Future research can be expanded in the following areas: To further elaborate the mechanism, metabolomics and ChIP-seq technologies can be combined to analyze whether the Wnt/β-catenin/c-MYC pathway simultaneously regulates other metabolic enzymes, thereby refining the signal-metabolism regulatory network. For intervention strategy exploration, the combined application of EVO and arginine depletion therapy can be explored, leveraging dual intervention (blocking endogenous synthesis + limiting exogenous supply) to enhance the inhibitory effect on CRC.

## 4. Materials and Methods

### 4.1. Reagents and Antibodies

EVO (T2868, HPLC ≥ 99.94%) and SKL2001 (T6989) were purchased from TargetMol Chemicals Inc. (Shanghai, China). Primary antibodies targeting multiple proteins, such as ASS1 (HA601101), β-catenin(ET1601-5), c-MYC(HA721182), and Cyclin D1 (HA721322), were purchased from HUABIO (Hangzhou, China). GAPDH (M20006), secondary antibodies (anti-mouse (M21001), and anti-rabbit (M21002)) were obtained from Abmart (Shanghai, China).

### 4.2. Cell Culture

The normal human colon cell line (NCM460), human colon cancer cell lines (HCT-116, HCT-8) and the murine colon cancer cell line MC38 were obtained from the American Type Culture Collection (ATCC, Manassas, VA, USA). These cells were nurtured in RPMI 1640 medium enriched with 1% penicillin–streptomycin (C100C5; NCM Biotech, Suzhou, China) and 10% FBS at 37 °C in a 5% CO_2_ incubator. For EVO treatment, EVO was prepared in DMSO, and the DMSO content in the culture medium was ≤0.1% (*v*/*v*) to avoid cytotoxicity. Cells were used during the exponential proliferation phase.

### 4.3. Cell Viability Assay

Cells (4 × 10^3^ cells/well) were seeded into 96-well plates and treated with EVO for 24, 48, or 72 h. After incubation, Cell viability was determined using the CCK-8 assay (CCK-8; Biosharp, Beijing, China).

### 4.4. Cell Colony Formation Assay

Cells (500 cells/well) were seeded in 6-well plates and treated with EVO as indicated. After incubation for two weeks, colonies were fixed with 4% paraformaldehyde and subsequently stained with a crystal violet solution (Beyotime, Shanghai, China).

### 4.5. EdU Assay

Cells (1 × 10^4^ cells/well) were seeded in 24-well plates and treated according to experimental groups. After 48 h of incubation in an incubator at 37 °C in 5% CO_2_, cells were subjected to labeling with 50 μM EdU for 2 h at 37 °C. Subsequent staining was carried out using the EdU Detection Kit (Beyotime, C0075S, Shanghai, China), with nuclei counterstained by Hoechst 33342, and fluorescence images were captured using a Fluorescence microscopy (Leica, Munich, Germany).

### 4.6. Wound-Healing Assay

To assess the ability of cells to migrate, a sterile 200 μL pipette tip was used to create a straight scratch once the cells in 6-well plates reached 90–100% confluence. Cells were then treated according to the experimental protocol and incubated in an incubator at 37 °C in 5% CO_2_. After 48 h, wound closure was visualized using an inverted microscope (Leica, Munich, Germany).

### 4.7. Measurement of Arginine and Citrulline Enzyme-Linked Immunosorbent Assay

Following the experimental design, cells and animal tissues in each group were treated accordingly. Cell culture supernatants and tissue homogenates collected from each group were centrifuged, aliquoted, and stored at −80 °C prior to ELISA analysis. Arginine and citrulline concentrations were measured using ELISA kits (Aifang Biological, Changsha, China). After completing all procedures as instructed by the kit manufacturer, absorbance was read at a wavelength of 450 nm.

### 4.8. shRNA-Mediated Gene Knockdown

The shRNA sequences targeting the target gene and plasmid synthesis were completed by Wuhan Miaoling Biotechnology Co., Ltd. (Wuhan, China) Subsequently, plasmid transformation and amplification were performed. After verification by bacterial liquid sequencing, lentiviruses were packaged by transfecting 293T cells with a three-plasmid system (psPAX2, pMD2.G, and the target plasmid) using polyethylenimine (PEI). The supernatant was collected at 48 h, filtered, and directly infected into target cells. With 2 μg/mL puromycin, stable clones were selected over a 7-day period. The effectiveness of the knockdown was confirmed using RT-qPCR and Western blot analysis, with blank and empty vector controls included to exclude nonspecific effects.

### 4.9. Real-Time Quantitative PCR (RT-qPCR)

Total RNA was isolated using TRIzol (Takara Bio, Kusatsu, Japan). Following homogenization, samples underwent chloroform extraction, isopropanol precipitation, and 75% ethanol wash. RNA concentration and purity were determined by spectrophotometry (A260/A280 and A260/A230 ratios). For cDNA synthesis, the Evo M-MLV RT Mix Kit with gDNA Eraser (Accurate Biotechnology, Wuhan, China) was used to remove genomic DNA contamination prior to reverse transcription. RT-qPCR was conducted with the SYBR Green Premix Pro Taq HS kit (Takara Bio, Kusatsu, Japan).

### 4.10. Western Blotting

Cells were lysed in RIPA buffer (with protease and phosphatase inhibitors), and protein levels were quantified via BCA Kit (Beyotime, P0012S, Shanghai, China). Following electrophoresis and subsequent transfer to PVDF membranes, the samples were subjected to blocking, then incubated with primary antibodies overnight at 4 °C, after which they were washed and exposed to secondary antibodies for 1 h. Finally, ECL reagent was used to visualize and image immunoreactive bands.

### 4.11. Immunofluorescence

Cells (2 × 10^4^/well) in the exponential phase were seeded in 24-well plates and treated as indicated. After 48 h, cells were fixed with 4% paraformaldehyde, permeabilized, and blocked. Samples were incubated overnight at 4 °C with primary antibodies and subsequently treated with fluorescent secondary antibodies for 1 h at room temperature in the dark. Nuclei were stained with Hoechst 33342.

### 4.12. Syngeneic Tumor Allograft Model

Approval for the animal experiments was granted by the Institutional Animal Care and Use Committee at Chongqing Medical University. C57BL/6J mice (Male, 6 weeks old) were provided by Hunan Slack Jingda Experimental Animal Co., Ltd. (Changsha, China) Mice were randomly assigned to two groups, and subcutaneously injected with 2 × 10^6^ MC38 cells into the right axillary region. After tumor establishment, the control group received vehicle (normal saline), while the treatment group was administered EVO (10 mg/kg) by oral gavage every 48 h. After 28 days, the mice were euthanized, with their tumors excised, weighed, and processed for additional tests.

### 4.13. Hematoxylin and Eosin Staining (HE)

The tissues from the tumor were preserved in a 10% neutral buffered formalin solution for 1 d, subsequently embedded in paraffin, and then sectioned. Sections were stained using a H&E staining kit (Solarbio, Beijing, China).

### 4.14. Immunohistochemistry (IHC)

Paraffin sections of tumor tissues were heated at 60 °C for 1 h, dewaxed in xylene, and rehydrated through graded ethanol. Antigen retrieval was performed by microwaving sections in pH 6.0 sodium citrate buffer for 4 min. After blocking, sections were placed at 4 °C and incubated overnight with the primary antibody, then they underwent incubation with the secondary antibody. Sections were stained for immunoreactivity with DAB-H_2_O_2_, counterstained with hematoxylin, then dehydrated, cleared using xylene, and mounted in neutral resin.

### 4.15. Molecular Docking

The structures of EVO and β-catenin were retrieved from PubChem (https://pubchem.ncbi.nlm.nih.gov) and the RCSB Protein Data Bank (PDB, https://www.rcsb.org/), respectively. Docking grids were created and converted into “PDBQT” format with PyMOL software (Version 3.1.0). Interactions between EVO and β-catenin were computed via AutoDock Vina (Version 1.2.5). Resulting three-dimensional structures were visualized with PyMOL.

### 4.16. Statistical Analysis

Statistical analyses were conducted utilizing GraphPad Prism 10.1.2 (GraphPad Software, Boston, MA, USA). Two-group comparisons were conducted utilizing a two-tailed Student’s *t*-test. For multiple comparisons, either one-way or two-way analysis of variance (ANOVA) was performed, followed by post hoc tests, specifically Tukey’s HSD for equal variance or Dunnett’s T3 for unequal variance; Sidak/Dunnett’s correction was applied for targeted pairwise comparisons to minimize type I errors. Statistical significance was defined as * *p* < 0.05, ** *p* < 0.01, and *** *p* < 0.001; comparisons marked as “ns” indicated no statistical significance.

## 5. Conclusions

In summary, our research highlights that the Wnt/β-catenin/c-MYC/ASS1 signaling pathway serves a key role in EVO-driven suppression of CRC, validated through In vitro and In vivo studies. This is the first study to identify that EVO exerts anti-CRC effects by inhibiting the Wnt/β-catenin/c-MYC pathway to specifically target ASS1, the rate-limiting enzyme in arginine synthesis, thereby inhibiting intracellular arginine metabolism. Our findings indicate that ASS1 shows potential as a viable therapeutic target for treating CRC and serves as a dependable marker linked to metabolic reprogramming in CRC; meanwhile, EVO exhibits great potential as a natural-derived agent for CRC therapy by integrating metabolic intervention and signaling pathway regulation.

## Data Availability

Data is contained within the article.

## Abbreviations

The following abbreviations are used in this manuscript:

CCK-8	Cell-Counting Kit-8
ASS1	Argininosuccinate Synthase 1
EVO	Evodiamine
CRC	Colorectal Cancer
IC_50_	Half maximal inhibitory concentration
SKL2001	Wnt/β-Catenin Pathway Agonist SKL2001
PBS	Phosphate-Buffered Saline
RPMI 1640	Roswell Park Memorial Institute 1640 Medium
